# Health Implications of Radon Exposure Among Children: A Systematic Review

**DOI:** 10.3390/children13020208

**Published:** 2026-01-31

**Authors:** Rasaq Yusuf, Phoka C. Rathebe

**Affiliations:** Faculty of Health Sciences, Department of Environmental Health, University of Johannesburg, Doornfontein Campus, P.O. Box 524, Johannesburg 2006, South Africa; powerray2003@yahoo.com

**Keywords:** radon, lung cancer, leukaemia, biomarker

## Abstract

**Highlights:**

**What are the main findings?**

- Childhood radon exposure is linked to lung cancer, as evidenced by adult dose–response cohorts indicating a projected risk increase of 10–20%.
- Some measurable inflammatory markers, cytogenetic changes, and epigenetic reprogramming were identified as early indicators of radon-induced biological stress in children.

**What are the implication of the main findings?**

- Early exposure functions as a latent trigger within the multistage carcinogenic progression of lung cancer, consistent with radiation epidemiology models where the incidence rate correlates with the early cumulative dose.
- Biological responses to radon exposure occur significantly prior to the clinical manifestation of malignancy, indicating that alpha-particle radiation induces a continuum of molecular injury that bridges disease onset. This highlights the importance of incorporating molecular biomarkers into radon exposure risk assessment frameworks.

**Abstract:**

**Background**: Radon exposure has been recognised as a risk factor for developing lung cancer and other health issues. The mutagenic changes associated with long-term radon exposure take 10–30 years to manifest, which may lead to a lower observed incidence of lung cancer in children. Children are more vulnerable to radon exposure and its effects due to their smaller lung capacity and faster breathing rates, resulting in greater radon inhalation. **Objective**: The aim of the study is to present current evidence on the association between radon exposure and health effects among children. **Methodology**: We conducted a systematic review of the available literature on radon exposure and its health impacts, focusing on children. A preliminary literature scoping was conducted in CINAHL, PubMed, Google Scholar, and ScienceDirect. Some of the search terms included: “children” OR “health” OR “implications” OR “radon” OR “exposure”. Subsequently, a comprehensive search was conducted, covering quantitative studies in EBSCOhost across all selected databases. The review adhered to the 27-item PRISMA (Preferred Reporting Items for Systematic Reviews and Meta-Analyses) checklist. The quality of the evidence gathered was assessed using the Grading of Recommendations Assessment, Development and Evaluation (GRADE) tool. The study was registered with PROSPERO under the ID: CRD420251269394. The review analysed 26 studies, all published between 1994 and 2025. **Results**: The incidence of lung cancer was projected to increase with childhood radon exposure, with statistical significance (OR per radon 100 Bq/m^3^ = 1.16; 1.05–1.31). Certain biological markers were associated with childhood long-term radon exposure: IL-5 (13.4%; 95% CI: 0.4–2.8; *p* = 0.044). **Conclusions**: Childhood radon exposure, although rarely enough to cause overt malignancy, contributes cumulatively to lifetime lung cancer risk and causes detectable biological markers.

## 1. Introduction

Radon is an invisible, odourless, and tasteless radioactive gas, and it is generated through the radioactive decay of parent compounds like radium, thorium, and uranium, present in soil and rocks [1]. The three predominant isotopes of radon are radon (^222^Rn), thoron (^220^Rn), and actinon (^219^Rn), which originate from the decay of uranium isotopes (^238^U, ^236^U, and ^235^U) [2,3], with half-lives of 3.82 days for radon, 55.8 s for thoron, and 3.98 s for actinon [2]. Radon isotopes are not inherently harmful; however, their decay products, such as polonium, bismuth, and lead, pose significant health risks [4]. These decay products are solid radioactive elements that emit alpha (α), beta (β), and gamma (γ) particles, with alpha particles tending to adhere to lung epithelium. The interaction of alpha particles with pulmonary parenchyma has the potential to damage DNA and consequently lead to lung cancer [5]. Among non-smokers, chronic exposure to radon remains the primary cause of lung cancer, and it is classified as the second leading cause among smokers [6]. The onset of lung cancer resulting from prolonged radon exposure may not be immediately apparent in children, except for asthma-related symptoms such as chronic cough, breathlessness, hoarseness, chest pain, and certain respiratory illnesses [7]. Similarly, chronic exposure to radon increases the risk of leukaemia in both children and adults [8,9].

Children are at high risk of exposure, particularly at home, school, and within their proximal environment. However, locations such as residences and educational institutions are intended to serve as safe environments where children can learn, reside, and engage in recreational activities free from hazardous substances. The probability of radon exposure is twice as high in children compared to adults due to their increased respiratory rate [10]. Beyond the residence, schools constitute another setting where children spend a considerable amount of time, thereby amplifying potential radon exposure. Understanding the relationship between increased radon exposure and its health effects is essential to mitigate the respiratory disease burden among the paediatric population. Consequently, the European Union’s environmental legislation recommends a maximum indoor radon concentration of 300 Bq/m^3^ [11]. Similarly, the South African National Nuclear Regulator (NNR) advocates a maximum indoor radon level of 300 Bq/m^3^ [11]. Currently, South Africa and other African nations lack statutory requirements for radon testing in educational institutions and residences [12].

The results on health outcomes associated with chronic radon exposure among children are inconsistent, suggesting a varying degree of statistical significance between the effects of radon exposure [13,14,15]. Furthermore, several studies have been conducted to ascertain the relationship between radon exposure and health risks in adult populations [16,17]. To document the evidence regarding the association between radon exposure among children and health effects, a systematic review was performed with a particular focus on both developing and developed countries. The review incorporated 26 studies that were published between 1994 and 2025. The quality of the evidence was evaluated using the Grading of Recommendations Assessment, Development and Evaluation (GRADE) tool. GRADE has proven to be an effective instrument for systematic reviews and healthcare guidelines, offering reliable evidence.

## 2. Materials and Methods

### 2.1. Study Design

The study’s design was grounded in the Population, Intervention, Comparison, and Outcome (PICO) framework. The PICO instrument enables a precise, systematic, and rigorous search for findings while delineating the components of quantitative health-related research [18,19]. The focus centred on prolonged radon exposure among children across various regions worldwide, including Low- and Middle-Income Countries (LMICs) and High-Income Countries (HICs), along with the associated health outcomes. A systematic review of scientific studies was conducted to examine the correlation by employing the PECO approach, emphasising the following points:

- Population (P): Children who are chronically exposed to radon in schools and homes.
- Exposure (E): chronic indoor radon exposure.
- Comparison (C): children who are not chronically exposed to indoor radon or exposed to low radon levels.
- Outcome (O): lung cancer, leukaemia, inflammatory biomarkers, and other health-related outcomes.

### 2.2. Protocol and Registration

This systematic review was conducted and documented in accordance with the Preferred Reporting Items for Systematic Reviews and Meta-Analyses (PRISMA) guidelines [20,21]. The review adhered to the 27-item PRISMA checklist (Supplementary Materials). The study was registered with the University of Johannesburg Faculty of Health Sciences Research Ethics Committee (REC) under Clearance Number REC-2969-2024, as well as in the PROSPERO database under the identification number CRD420251269394.

### 2.3. Information Sources and Search Strategy

A preliminary scoping of literature was conducted utilising CINAHL, PubMed, Google Scholar, and ScienceDirect. The search incorporated terms such as: “children,” OR “health,” OR “implications,” OR “radon,” OR “exposure,” OR “low-middle income countries,” OR “outcomes,” OR “asthma,” OR “lung cancer,” OR “leukaemia,” OR “cough.” Subsequently, the researcher independently performed comprehensive searches of electronic databases, concentrating on accessible quantitative studies published between August 1994 and 25 August 2025, within EBSCOhost across all selected databases. This methodology aimed to identify studies on the health impacts of radon exposure among children in diverse regions globally. The search was confined to research articles published in English, and the identified references were uploaded to RAYYAN [22], a cloud-based platform designed for researchers undertaking systematic reviews and meta-analyses.

### 2.4. Studies’ Selection and Data Extraction Processes

#### 2.4.1. Study Selection and Data Extraction

This review considered eligible studies that employed quantitative methodologies to investigate the relationship between radon exposure in children and associated adverse health effects. Chronic radon exposure was defined as exposure lasting three months or longer, occurring at either home and/or school. The scope of the search was expanded to include studies conducted beyond South Africa, given that this was the first review of its kind within the region. Inclusion criteria encompassed studies that: (a) exclusively focused on chronic radon exposure among children in school and/or home environments and the associated health consequences; (b) directly compared cases, children diagnosed with health conditions such as lung cancer, leukaemia, lymphoma, or exhibiting elevated inflammatory biomarkers, who experienced continuous radon exposure, with controls, children not exposed to radon or exposed to minimal levels; (c) assessed whether children exhibited specific health conditions in conjunction with ongoing radon exposure or demonstrated improvement following reduced exposure; (d) utilised quantitative research designs, including case–control, cohort, cross-sectional, and longitudinal studies. There was no restriction regarding the presentation of results. Accepted metrics included percentages, risk ratios, odds ratios, and hazard ratios. Studies were excluded if they involved participants over 18 years of age, or if they employed qualitative or mixed-methods designs, as these criteria conflicted with the protocols of the research.

#### 2.4.2. Search Results

**Figure 1:**
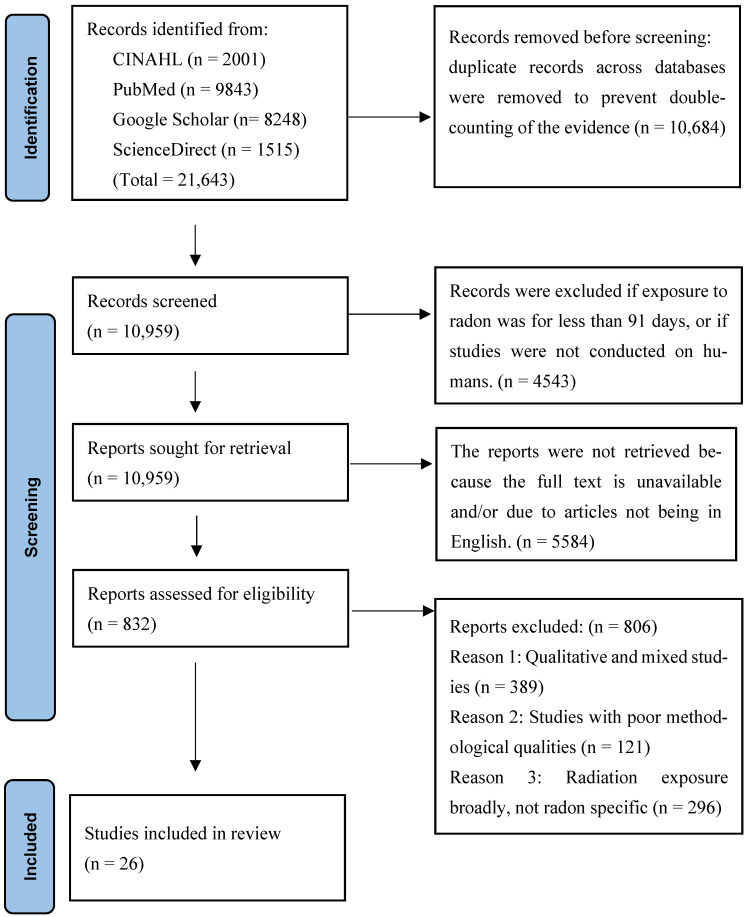
PRISMA Flow Chart on Literature Search.

An initial search was undertaken on EBSCOhost to find studies conducted between 1994 and 25 August 2025, using the keyword “children” (representing the sample population). The search yielded 21,643 results with low sensitivity [23]. Subsequently, additional details pertaining to the phenomenon of interest, “radon exposure,” were incorporated, reducing the number of relevant research articles to 10,959. Further refinement was achieved by adding the term “health outcomes, “ which decreased the results to 6416. Limiters such as full-text availability, references, peer-reviewed status, quantitative articles published in English, and studies involving participants with health-related problems were then applied. The search terms: “children,” “radon,” “exposure,” and “health outcomes,” were connected by the Boolean operator ‘OR,’ resulting in a total of 832 articles. These retrieved articles were subsequently examined manually, with a focus on the health impacts of chronic radon exposure among children. The search themes were then combined with the Boolean operator “AND,” following the same systematic approach. The final corpus encompassed 26 articles, as presented in Table 1. In summary, the search process utilising EBSCOhost and other methodologies adhered to the PRISMA Flow Chart on Literature Search outlined in Figure 1.

#### 2.4.3. Rating the Quality of Evidence

The researcher employed the principles of the Grading of Recommendations Assessment, Development and Evaluation (GRADE) to evaluate the quality of evidence pertaining to the health effects of indoor radon exposure among children [44]. The assessment of evidence quality predominantly depends on study design and sample size, with randomised controlled trials (RCTs) regarded as the gold standard. However, RCTS are rarely conducted in environmental health research due to ethical considerations and the vulnerability of the paediatric population [47,48]. Consequently, this review primarily relied on observational studies to synthesise existing literature. This approach necessitated the application of a modified GRADE methodology, incorporating observational study designs such as cohort, case- control, ecological, cross- sectional, and longitudinal studies. To appraise the quality of evidence, the researcher accounted for additional factors including sample size, confounding variables, and potential biases, as detailed in Table 2. The influence of these factors on evidence quality is documented in scholarly literature [49,50]. The association between radon exposure in children and related health outcomes was further examined in the identified observational studies, justified by their credibility in assessing the quality of evidence necessary to establish a link between environmental exposure and disease [51,52,53]. Such studies (cohort, case- control, cross- sectional, and longitudinal) demonstrated results comparable to RCTs, indicating their significance in academic research [52,53]. The reliability of exposure assessment was ascertained by evaluating the quality of the included studies using the Newcastle- Ottawa Scale (NOS) for cohort, case–control, and cross- sectional studies. The aggregate NOS score ranges from 0 to 9 for cohort and case–control studies, and from 0 to 10 for cross- sectional studies. Studies attaining an overall NOS score of at least 6 (or a minimum of 7 for cross- sectional studies) were classified as “high quality”. Those with a score of 4–5 (or 5–6 for cross- sectional studies) were deemed “moderate quality”, while scores below this threshold were categorised as “low quality” [54], as outlined in Table 2.

#### 2.4.4. Risk of Bias Among Selected Studies

In this review, the researcher conducted a critical evaluation of included studies to identify potential biases by examining research methodologies, such as those employed to formulate the research questions, sample sizes, inclusion and exclusion criteria, tools used to measure radon levels, duration of radon measurements, and the methods of data analysis. Studies that failed to account for confounding factors when assessing the health effects of radon exposure were excluded. The search strategy included studies from 1994 to 2025, acknowledging the limited publication of radon research among children, with the aim of minimising bias associated with a restricted literature search period. Furthermore, the fundamental principle of a systematic review is a collaborative academic effort that synthesises qualitative evidence across a substantial sample [55,56]. The selected studies underwent peer review by two senior researchers. Due to the scarcity of evidence from LMICs, the researcher faced limitations owing to the small number of available articles, which impeded the possibility of conducting a meta-analysis. This limitation may introduce potential bias into the evidence synthesis presented in this systematic review. Nevertheless, various statistical techniques, including percentages, risk ratios, odds ratios, hazard ratios, confidence intervals, and *p*-values, were employed to compile the findings of this review.

## 3. Results

### 3.1. Health Implications of Radon Exposure in Children

#### 3.1.1. Lung Cancer

The PRISMA guidelines were adhered to when conducting this systematic literature review. The results identified 24 studies, with data derived from seven publications, which were extrapolated to investigate the relationship between radon exposure and lung cancer in children. Studies examining this association are shown in Table 3.

The BEIR VI team [24] studied miner cohorts, encompassing approximately 68,000 workers and 2700 lung cancer mortality. A dose–response framework was established using the excess relative risk (ERR) model.

ERR = 0.025 y(a) (W_1_ + ½ W_2_),

where y(a) indicates age adjustment, and W_1_ and W_2_ are the cumulative exposures during previous and earlier time periods.

The pooled ERR was estimated at 0.005 (95% CI: 0.002–0.010), which corresponds to approximately a 16% increase in lung cancer risk for every 100 Bq/m^3^ of exposure, with an additional 10–20% increase in relative risk anticipated for childhood exposure.

In a comprehensive European case–control study involving 7148 cases and 14,208 controls across 13 different studies [25], a direct linear relationship was identified between indoor radon levels and lung cancer risk. The odds ratio (OR) for each 100 Bq/m^3^ increase in radon was 1.16, with a 95% confidence interval of 1.05 to 1.31, indicating an approximate 16% increase in lung cancer risk per 100 Bq/m^3^. Similarly, a pooled analysis conducted in North America [26], which included 3662 cases and 4966 controls, observed an OR of 1.11 (95% CI: 1.00–1.28) per 100 Bq/m^3^ after adjusting for smoking and other confounding variables. These findings are in close agreement with the European data [25].

Meta-analysis [29], which integrated case–control studies conducted across Europe and North America at exposure levels ranging from 25 to 300 Bq/m^3^, identified a mean Excess Relative Risk (ERR) of 0.14 (95% Confidence Interval: 0.01–0.29) per 100 Bq/m^3^. This meta-estimate offered quantitative corroboration of a linear exposure–response relationship and impacted the parameterization of both the BEIR VI and WHO models.

**Figure 2:**
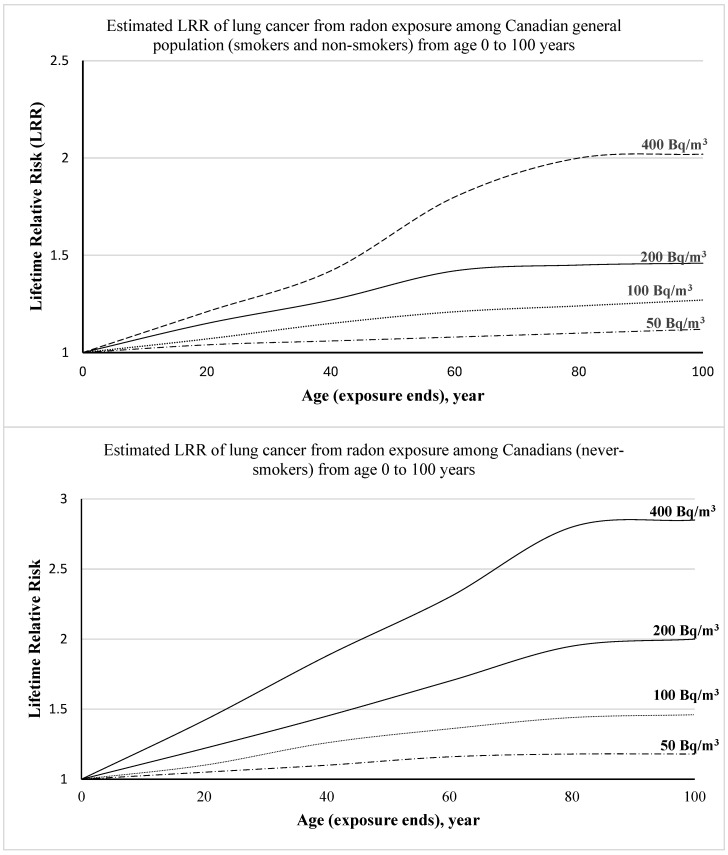
LRR of lung cancer due to radon exposure from birth to 100 years old [27].

In 2013, Chen [27] used the national residential exposure data to model the lifetime risk of lung cancer within the Canadian population, applying BEIR VI coefficients. At a concentration of 400 Bq/m^3^, the estimated lifetime relative risk for smokers and non-smokers aged 15 ranged from 1.2 to 1.4, indicating that early-life exposure could potentially increase the lifetime risk of lung cancer by up to 20%, as further demonstrated in Figure 2 (created using the data from ref. [27]). An additional study also examined 18,971 Canadian households [30], with a geometric mean radon level of 108.2 Bq/m^3^. This research estimated that younger populations receive approximately 1.4 times higher lifetime radon doses compared to individuals born in 1950, taking into account housing characteristics and occupancy patterns.

A systematic review [28] analysed eight Chinese case–control studies involving 8200 lung cancer cases and 18,500 controls. The observed age-dependent excess relative risk (ERR) indicates a 1.2-fold increased risk of lung cancer in individuals exposed to radon during childhood compared to those exposed during adulthood (95% CI: 0.9–1.6), although this finding did not attain statistical significance.

#### 3.1.2. Leukaemia

In accordance with the Preferred Reporting Items for Systematic Reviews and Meta-Analyses (PRISMA) guidelines, a systematic literature search was conducted. This search yielded 34 studies, of which eleven focused explicitly on the connection between paediatric radon exposure and childhood leukaemia. A comprehensive list of identified studies examining the association between radon exposure and childhood leukaemia is presented in Table 4. Eligible studies examined radon exposure among children at home, during school terms, or during hospital stays. Radon exposures were measured using various methods, including short-term measurements, geocoding, prediction models, and alpha-track detectors for long-term assessments.

Independent case–control studies involving more than 900 children (cases and controls), assessed long-term radon exposure using alpha track detectors [31], with radon exposure levels ≥ 148 Bq/m^3^, yielding a relative risk (RR) of 1.02 (95% CI 0.5–2.0), and [32], with radon exposure > 100 Bq/m^3^, producing an odds ratio (OR) of 1.1 (95% CI 0.6–2.0). Both studies concluded with similar outcome; there was no association between radon exposure and childhood leukaemia. Similarly, in Norway [37], a longitudinal study spanning 40 years involving over 700,000 children found no significant correlation between radon exposure (≥100 Bq/m^3^) and leukaemia, with hazard ratios (HR) approximately equal to 1.00 (95% CI 0.87–1.15). It is noteworthy that study [37] acknowledged the crude nature of the exposure assessment, which utilised buffered values for numerous dwellings instead of direct measurements. Furthermore, international health agencies, including the International Agency for Research on Cancer (IARC) and the World Health Organisation (WHO), have indicated that the evidence linking radon exposure to childhood leukaemia remains limited, particularly when contrasted with the well-established association between radon and lung cancer [58,59,60].

In contrast, reference [35] modelled cumulative radon exposure among 2400 cases, finding a positive association with rate ratios of 1.63 for high cumulative exposure. Furthermore, Ref. [57], in Texas, United States, hypothesised a marginal increase in the incidence of lymphoma associated with radon exposure, although with overall inconsistencies. The findings of [35,57] could be interpreted as outliers, again contrary to the harmonised European pooled analyses of case–control datasets, which showed no associations or dose–response signals between radon exposure and childhood leukaemia [34].

#### 3.1.3. Biomarkers

Table 5 outlines studies that explored radon-induced biomarkers. The findings encompassed eleven studies, with data from eight publications extracted to analyse the association between radon exposure and biological markers (biomarkers) in children, as detailed below. Radon exposure in paediatric populations has been correlated with observable alterations in biomarkers, including elevated inflammation markers (CRP, IL-1β, IL-5 in asthmatics) and epigenetic modifications (methylation) [61,62,63].

In a cross-sectional study involving 68 youths aged 6–14 years, Ref. [40] observed that indoor radon concentrations were positively correlated with salivary C-reactive protein (CRP; β = 0.31, *p* = 0.007) and interleukin-1β (IL-1β; β = 0.33, *p* = 0.016), thereby indicating systemic inflammation. Similarly, in a cohort of 299 schoolchildren with asthma [7], it was demonstrated that there was a 13.4% (95% CI: 0.4–28.0%; *p* = 0.044) increase in IL-5 levels associated with each increment in estimated monthly radon exposure, suggesting T_H_2-mediated immune modulation in response to radon.

Epigenetic effects were evaluated in the longitudinal ALSPAC cohort [41], which included 786–980 children with complete data at three time points (birth, age 7, and 17). The study identified exposure-related DNA methylation at cg16451995 (at birth) and cg01864468 (at age 7), indicating that early-life radon exposure may affect DNA methylation patterns in later childhood.

Cytogenetic and genotoxic findings were also reported in two European studies. In Slovenia, Ref. [42] studied 85 schoolchildren (aged 9–12) exposed to exceptionally high indoor radon levels (≥7000 Bq/m^3^; annual effective dose 7–11 mSv) and observed increased chromosomal aberrations (4% vs. 2.5% in controls exposed to <400 Bq/m^3^). In Poland, Ref. [43] examined 94 residents from Kowary City. They found that residential radon levels, measured with CR-39 alpha-track detectors, were associated with increased DNA damage in the comet assay, but this association was not statistically significant when correlated with γH2AX staining due to rapid loss of the γH2AX signal.

## 4. Discussion

### 4.1. Childhood Radon Exposure and Lung Cancer

The evidence confirms that residential radon exposure causes cancer in adults, but its impact on children remains less certain. Radon decay products emit alpha particles that can induce DNA double-strand breaks and mutagenic damage in bronchial epithelial cells [64,65]. However, directly linking this mechanism to a measurable risk for childhood lung cancer is challenging due to the rarity of cases, long latency periods, and potential exposure misclassification. Nonetheless, models and estimated risk assessments consistently suggest that early-life radon exposure could result in a slight yet biologically significant increase in lifetime lung cancer risk [24,26].

Despite the biological plausibility, empirical data directly linking childhood radon exposure to lung cancer remain limited. Consequently, epidemiological evidence is predominantly derived from cohorts of adults and occupational miners. In the BEIR VI [24] report, data were modelled from over 68,000 miners, establishing a nearly linear dose–response relationship, with an Excess Relative Risk (ERR) of approximately 0.025 per working-level month. This translates to a 10–16% increase in lung cancer risk per 100 Bq/m^3^ of radon exposure. The modelling datasets suggest that exposures during early life could augment lifetime risk by an additional 10–20%, primarily attributable to cumulative dose accumulation and extended latency periods, rather than solely increased biological radiosensitivity [25,26].

Epidemiological studies in the general population [25,26], although not explicitly concentrated on the paediatric age group, reveal a linear, no-threshold risk pattern at residential exposure levels. Subsequent simulations [27,30] enhanced projections by incorporating variables such as age, fluctuating radon concentrations, and housing trends, indicating that contemporary children, who spend increased time indoors in well-insulated dwellings, may accrue higher radon doses than previous generations. In the Chen age-specific model [27], the lifetime relative risk (LRR) of paediatric lung cancer was estimated to be between 1.2 and 1.4 for children exposed to radon at 400 Bq/m^3^ from birth through adolescence. This projection corroborates computational analyses that suggest the significance of extended exposure and the enduring biological effects of early insults [30].

Direct observation of paediatric lung cancer caused by radon is nearly nonexistent, with estimates below 0.3% of all lung cancers [58]. This rarity reflects both the genuine rarity of the disease and the methodological difficulties in measuring lifelong radon exposure. Case–control studies in children lack statistical power due to few cases, and cohort studies would require decades of follow-up to detect the disease [59]. Consequently, the most reliable approach is to use current estimates based on hybrid models combining miner data, adult case–control studies, and age-adjusted dose–response coefficients [24,25,27].

Current models converge on a risk gradient of approximately 10–16% increased lifetime lung cancer risk per 100 Bq/m^3^ of exposure, with early-life exposure adding an extra 10–20% proportional risk [26,27,30]. Biological mechanisms, such as genotoxic damage, persistent inflammation, and epigenetic modifications, support the validity of these estimates [41,64]. The next step involves addressing the knowledge gap by prioritising longitudinal, biomarker-based studies across various paediatric populations to determine dose–response relationships and validate the existing model assumptions [59,65].

### 4.2. Childhood Radon Exposure and Leukaemia

Epidemiological evidence examining the association between residential radon exposure and childhood leukaemia remains intricate, diverse, and considerably less conclusive than the well-established relationship between radon and lung cancer. Although alpha-particle emissions from radon progeny can potentially induce DNA double-strand breaks and chromosomal abnormalities in hematopoietic stem cells, translating this mechanistic potential into a detectable population-level effect in children has proven to be methodologically challenging [31,36].

Over more than thirty years of research, encompassing 34 identified studies with 11 explicitly concentrating on paediatric outcomes, findings have varied from no effect to modest positive associations and inconsistent results. Early investigations utilising direct household alpha-track detectors [31,32], which were among the most precise methodologies for measuring exposure, did not demonstrate a significant increase in leukaemia risk, even at concentrations exceeding 148 Bq/m^3^. These null findings were consistent across comprehensive population-based analyses, including the United Kingdom Childhood Cancer Studies [34,66] and a record-based case–control study [38], which incorporated national radon maps and detailed geocoding. Despite the increased statistical power afforded by tens of thousands of cases and controls, these studies found no dose–response relationship or spatial correlation between radon exposure and leukaemia.

However, certain studies in continental Europe have yielded some suggestive results. The Danish case–control study [35] reported a rate ratio of 1.63 for children in the highest cumulative exposure groups, and [57] similarly reported a slightly increased incidence of lymphoma in Texas, though with regional variations. The harmonised European pooled dataset [60], revealed a statistically significant but small positive association (pooled OR = 1.03 per 100 Bq/m^3^; 95% CI: 1.01–1.06). Although this association is modest, it aligns with a dose-dependent pattern consistent with effects from low-dose ionising radiation, especially considering long latency periods and cumulative exposures from in utero to early childhood [60].

However, the inconsistencies among studies highlight challenges in radon epidemiological studies. Firstly, exposure misclassification is prevalent; using area-level radon prediction maps, registry or short-term measurements often does not capture significant inside-home temporal fluctuations or different children’s occupancy patterns [38]. Radon levels in homes can vary seasonally and daily by more than tenfold, and such misclassification generally introduces biased results, potentially hiding small but genuine associations [62,67]. Secondly, many studies do not consider residential mobility, which is a crucial factor in paediatric populations, where total exposure largely depends on how long children remain in a particular residence early in life [37].

From a biological perspective, the proposed association between radon and leukaemia relies on whether alpha emissions originating from radon progeny can deliver a biologically relevant dose to the bone marrow microenvironment, where leukemogenic mutations develop [65]. Unlike the bronchial lining, the bone marrow receives a considerably lower absorbed dose from inhaled radon progeny, generally estimated at less than 1% of the dose received by the bronchial tissue [24]. Nevertheless, experimental evidence suggests that even low-dose alpha irradiation can induce clonal hematopoietic changes, oxidative stress, and epigenetic reprogramming associated with leukemogenesis [67]. Consequently, although the dose is minimal, the high linear energy transfer characteristic of alpha particles implies that even infrequent exposures may result in significant biological effects in individuals with genetic vulnerability [62].

Large-scale cohort studies, such as those conducted by [37] in Norway and [38] in Finland, have delivered some of the most statistically robust assessments to date. However, both investigations reported null results, primarily attributable to the indirect methodologies employed for exposure assessment. Reference [37] noted that utilising geospatially buffered exposure estimates, as opposed to direct indoor measurements, diminished the likelihood of detecting associations. Concurrently, newer exposure modelling techniques [38] that integrate geological and structural predictors have improved spatial resolution but continue to encounter challenges in reconstructing historical exposure levels, especially for older structures and diverse ventilation practices.

Within the broader context of radiation epidemiology, the overall evidence suggests that, if a causal effect exists, it is probably small and varies with exposure levels, mainly appearing at higher residential concentrations or in genetically predisposed subgroups [58,59]. These findings suggest that residual confounding, exposure misclassification, and ecological fallacies continue to complicate definitive causal interpretations [60]. From a public health perspective, the impact is subtle. Even a weak, yet genuine, association could have substantial effects at the population level, considering the widespread indoor radon exposure and children’s vulnerability in blood development. Nevertheless, until more high-quality, prospective studies with standardised exposure assessments are conducted, the evidence for a direct causal relationship between residential radon and childhood leukaemia remains limited or inconclusive [58,59,60].

### 4.3. Childhood Radon Exposure and Biomarkers

Over the past twenty years, there has been a growing interest in understanding how radon exposure might induce molecular and cellular alterations in children. This heightened susceptibility is attributed to their rapidly dividing cells, increased minute ventilation, and extended lifespan expectancy post-exposure, each contributing to an increased sensitivity to the biological impacts of ionising radiation [67,68]. Utilising the PRISMA framework, eight studies gave analysable data linking childhood radon exposure with specific biomarkers related to inflammation, genotoxicity, or epigenetic modifications (see Table 5). Although the evidence remains preliminary, it reliably indicates that even low to moderate levels of radon, those below occupational safety standards, can cause measurable biological responses in children [7,40].

Inflammatory biomarkers serve as highly sensitive indicators of early physiological responses to indoor radon. Exposure to higher radon levels [40] was found to be positively associated with increased salivary C-reactive protein (CRP; β = 0.31, *p* = 0.007) and interleukin-1β (IL-1β; β = 0.33, *p* = 0.016) in 68 children aged 6–14 years. These results suggest a systemic pro-inflammatory state, potentially resulting from low-level oxidative stress caused by radon progeny deposits in the airway epithelium. Similarly, a significant increase in interleukin-5 (IL-5; 13.4%, 95% CI: 0.4–28.0%, *p* = 0.044) was observed among 299 school-aged children with asthma [7]. This indicates that radon exposure may enhance T_H_2 immune responses and exacerbate inflammatory conditions within the airway. Overall, these findings provide biological evidence that radon may influence cytokine activity in children, particularly in those with pre-existing respiratory conditions.

Beyond inflammatory pathways, increasing evidence associates radon with epigenetic and cytogenetic disruptions. The ALSPAC study [41] provides a detailed dataset tracking over 900 children from birth to adolescence. It found consistent links between estimated residential radon exposure and DNA methylation changes at CpG sites cg16451995 (at birth) and cg01864468 (at age 7), supporting the hypothesis that early-life exposure results in enduring epigenetic reprogramming. These molecular modifications may have long-term implications for gene expression involved in cell cycle regulation, DNA repair, or immune function.

Children residing in environments with elevated radon levels have demonstrated signs of cytogenetic damage and DNA double-strand breaks. In a Slovenian case–control study [42], a 4% increase in chromosomal aberrations was observed in cases compared to 2.5% in controls, at indoor radon concentrations exceeding 7000 Bq/m^3^ (annual dose 7–11 mSv). This finding supports the hypothesis that high residential radon exposure can induce detectable genomic instability. Further corroborating this hypothesis, researchers [43] reported elevated levels of serum phosphorylated histone γH2AX among children exposed to moderate radon levels in Kowary City regions, Poland. This reinforces the biological plausibility of radon-induced genotoxic stress even among non-mining populations. Conversely, Ref. [45] noted the absence of published paediatric biomarker studies or sufficiently powered epidemiological research from Africa linking radon exposure to health outcomes. Their review advocates for the initiation of such studies and the adoption of standardised measurement methods.

While evidence indicates a spectrum of molecular effects, ranging from inflammation to DNA damage and epigenetic modifications, certain limitations remain. Many investigations were characterised by small sample sizes and indirect exposure assessments, with few establishing definitive causality over time [41,43]. Variability in biomarker results may also stem from co-exposures, indoor environmental factors such as tobacco smoke and particulate matter, as well as individual susceptibility [69,70]. Nevertheless, consistent findings across diverse populations endorse the hypothesis that radon exposure can cause measurable biological responses in children, aligning with models of alpha-particle induced oxidative stress and DNA damage [7,65].

Overall, this emerging body of research highlights the potential of biomarkers as early indicators of radon-induced biological stress in children. Future investigations should prioritise large-scale, longitudinal studies that incorporate multi-household biomarker surveys, including epigenetic, transcriptomic, and proteomic signatures, coupled with personal radon dosimetry. This methodology can assist in revealing the dose–response relationships and in identifying populations at high risk [7,40,43]. Such research would bridge the gap between molecular biomarkers and long-term disease risk, thereby advancing radon risk assessment beyond conventional epidemiological approaches.

## 5. Strengths and Limitations

The study synthesised evidence from existing literature across epidemiological and modelling fields in various geographical regions [24,44,45,65]. The effort was supported by linking miner cohort data with population-based and paediatric-focused studies, creating a logically coherent narrative that places biological plausibility alongside empirical and theoretical evidence [25,31,64]. This approach enhanced causal inference despite limited data on the association between childhood radon exposure and health effects [65]. The review demonstrated a link between alpha-particles and the modelled lung cancer risks by maintaining dose–response effects among the paediatric population [24]. Additionally, there was alignment between observed biological markers, DNA damage, and interleukins, which underpins the biological plausibility of the review. The study assesses both null and positive findings equally and offers a cautious interpretation of weak or inconsistent associations (leukaemia), consistent with the WHO and UNSCEAR’s cautious conclusions [37,59,65].

The main limitation of this study is its dependence on extrapolated data derived from cohorts of miners and adult case–control studies [26,31]. Although this methodology appears credible due to the infrequency of paediatric lung cancer cases, the study presumes uniform dose–response effects across all age groups, which may result in an overestimation of biological comparability [24]. Certain epidemiological studies referenced employ indirect or modelled radon exposure estimates rather than direct longitudinal indoor measurements, potentially leading to exposure misclassification and non-differential errors [32]. Although the examined literature has a global scope, it is predominantly weighted towards high-income and temperate countries. There is a paucity of research originating from Africa, South Asia, and Latin America- regions where building materials, ventilation practices, and uranium-rich soils may significantly influence exposure profiles and health outcomes [1,45]. This underrepresentation indicates the necessity for additional research in these regions. It is incumbent upon regional researchers to address these gaps to attain a more comprehensive understanding.

## 6. Conclusions

The review offers a comprehensive overview of childhood radon exposure and its potential health effects. The causal relationship between alpha particles and lung cancer remains predominantly theoretical but biologically credible, supported by dose–response cohorts derived from adult populations [24,65]. Although evidence concerning childhood leukaemia is inconclusive owing to limited population-based studies and uncertainties, the evidence correlating radon exposure with biomarkers appears promising. It demonstrates measurable inflammatory markers, cytogenetic alterations, and epigenetic reprogramming.

Essentially, the collective evidence suggests that radon exposure during childhood, although rarely enough to cause overt malignancy, contributes cumulatively to lifetime lung cancer risk and causes detectable biological markers even at indoor exposure levels below regulatory limits [59,65]. To objectively confirm the link between radon exposure and childhood health effects, large-scale, short-term, and long-term studies with personal dosimetry are needed, while accounting for confounding factors [45,69]. Until then, we must continue to support health promotion efforts to reduce indoor radon exposure, especially in environments occupied by children.

## Figures and Tables

**Table 1:** The 26 studies included in the review identified with the modified GRADE system.

Study, Year	Study Domain	Strength of Evidence	Main Outcome
BEIR VI [24]	Cohorts of miners modelled for lung cancer risk	High	Risk modelled for radon-lung cancer dose–response with no direct paediatric data.
Darby et al. [25]	A case–control study modelled for lung cancer risk	High	Linear exposure-response risk, adopted in the WHO guidelines
Krewski et al. [26]	Case–control study, a projection for lung cancer risk	High	Aligned with the BEIR VI projection
Chen [27]	Quantitative survey for lung cancer risk projection	Moderate–high	Estimates of lifetime radon-lung cancer risks with varying parameters-age, gender, radon levels
Su et al. [28]	Systematic review of adult lung cancer cases	High	Projected ERR 1.2 more than adult ERR (95% CI 0.9–1.6)
Lubin et al. [29]	Meta-analysis of lung cancer risk projection	High	Pooled analysis established a low-risk projection
Simms et al. [30]	Modelling of cohorts for lifetime exposure risk	Moderate	A 1.4 times risk for cumulative radon exposure in the paediatric age group
Lubin et al. [31]	Case–control for radon exposure vs. risk of leukaemia	Moderate–high	No significant association of risk of leukaemia and childhood radon exposure
Steinbuch et al. [32]	Case–control exposure risk for paediatric leukaemia	Moderate–high	Non-significant association in a methodologically underpowered study- cases vs. controls
Kaletsch et al. [33]	Case–control exposure risk for paediatric leukaemia	Moderate–high	Null results with uncertainty
UKCCS Investigators [34]	A national case–control study explored childhood exposure to leukaemia risk	High	Comprehensive data with an overall null effect
Raaschou-Nielsen et al. [35]	An extensive participant-based case–control modelled exposure risk	High	Cumulative childhood exposure with a significant leukaemia risk
Kendall et al. [36]	Record-based case–control with a large participants	Moderate	Null association, possibly a record attrition and misclassification
Del Risco Kollerud et al. [37]	Longitudinal study of large paediatric cohorts over 40 years	High	Overall, not statistically significant (*p* > 0.05)
Hauri et al. [15]	Population-based cohorts of paediatric leukaemia cases	Low–moderate	Exposure not linked with the regional childhood leukaemia trends
Peckham et al. [35]	Ecological-based registry	Low–moderate	Low exposure risk observed
Nikkila et al. [38]	Exposure prediction modelling	Low–moderate	No significant association, possibly due to methodological heterogeneity
Yoshinaga et al. [39]	Preliminary report	Low	No observed association, contributed by sparse data and low magnitude effects
Taylor et al. [40]	Cross-sectional review of exposure with associated inflammatory biomarkers	Moderate	Significant levels of paediatric biomarkers associated with radon exposure
de Vocht et al. [41]	DNA methylation correlations with exposure among paediatric cohorts	Moderate	Biological marker linked with radon exposure
Banzon et al. [7]	A prospective paediatric biomarker cohort study	Moderate–high	Biomarker response-dependent dose concentration association
Bilban et al. [42]	Biomonitoring of cases and controls in school children	Moderate–high	Assay of chromosomal damage linked with radon exposure
Walczak et al. [43]	Population-based cohorts, including paediatric cohorts	Moderate–high	DNA damage associated with alpha-particle exposure is consistent with the literature. Exposure level studied objectively with CR39
Mphaga et al. [44]	A cross-sectional field study	Low–moderate	The association of biological markers with radon exposure was not primarily explored
Rathebe et al. [45]	Systematic review focused on African countries	High	Integrates African literature and identifies the paucity of biomarker studies within the continent
Tunisia Radon Survey [46]	Cross-sectional study of radon exposure	Low–moderate	Focused mainly on indoor radon concentration levels with no health implication endpoints

**Table 2:** Factors influencing the quality of evidence review.

Study sample size ≥ 300	Study sample size ˂ 300
Consistent findings	Conflicting findings
Confounders controlled	Confounders not controlled
Cohort, case–control, panel studies	Cross-sectional studies
Long duration of indoor radon measurement, ≥90 days	Short indoor radon measurement, ˂90 days or where radon geological data utilised

**Table 3:** Childhood radon exposure and lung cancer.

Author, Year	Data Collection	Radon Exposure (Bq/m^3^)	Model Criteria	Result (95% CI)	Projected Risks of Lung Cancer
BEIR VI [24]	Pooled 11 cohort studies- 68,000 miners and 2700 lung cancer deaths.	Estimated radon exposure- 100 to ≥1000 Bq/m^3^	Excess Relative Risk (ERR) = 0.025 y(a) (W1 + ½ W2)	*y*(a) = age-specific adjustment to the RR; W1 = cumulative exposure received 5–15 y before age a; W2 = cumulative exposure up to age a-15. (95% CI 0.002–0.010)	Projected increase of 16% risk of lung cancer in adults per 100 Bq/m^3^ radon exposure. An additional 10–20% RR due to childhood exposure.
Darby et al. [25]	Analysis of pooled 13 case–control studies from Europe- 7148 cases, 14,208 controls.	0–400 Bq/m^3^	Linear ERR model without threshold.	OR per 100 Bq/m^3^ = 1.16 (1.05–1.31)	Lung cancer risk increased by approximately 16% per 100 Bq/m^3^. The basis for lifetime risk is due to early exposure.
Krewski et al. [26]	Combined analysis of North American case–control study-3662 cases, 4966 controls	Mean exposure- 91 Bq/m^3^; range, 0–300 Bq/m^3^.	Smoking and other confounders were adjusted for.	OR per 100 Bq/m^3^ = 1.11 (1.00–1.28)	Similarly to Darby et al., the basis for Canadian childhood exposure risk modelling.
Lubin et al. [29]	Meta-analysis of 8 case–control studies from Europe, and North America	Low conc. 25–300 Bq/m^3^	Utilised the weighted ERR model for the 8 studies for precision.	Average of 0.14 per 100 Bq/m^3^ radon exposure; (95% CI 0.01–0.29)	Suggests a positive linear trend, with a 14% increased risk; the study contributed to the input for the BEIR VI and WHO models.
Chen [27]	A modelling study extrapolated from the Canadian residential data and the BEIR VI model.	Ranges from 50 to 400 Bq/m^3^	Lifetime lung cancer risk projection with focus on age, gender, smokers/non-smokers.	Lifetime relative risk for cohort population at age 15 years exposed to 400 Bq/m (passive smokers and non-smokers) 1.2–1.4	Childhood exposure was associated with up to 20% risk of lung cancer.
Simms et al. [30]	Demographic model for 18,971 households.	Geometric mean- 108.2 Bq/m^3^	Computational model based on the households and BEIR VI coefficients.	Estimates a 1.4 increase in the lifetime dose of the younger population compared to the 1950 birth cohorts.	Probably, the younger population is currently accumulating a greater lifetime radon dose due to housing patterns.
Su et al. [28]	Systematic review of 8 studies in China, 8200 lung-cancer cases, 18,500 controls.	Average exposure 55 Bq/m^3^, range 15–250 Bq/m^3^	Prediction with meta-regression ERR vs. age of radon exposure	Projected ERR 1.2 more than adult ERR (95% CI 0.9–1.6)	A marginal increase in age dependence, not statistically significant.

**Table 4:** Childhood radon exposure and leukaemia.

Author, Year	Country	Study Design	Exposure Assessment Method	Main Results
Lubin et al. [31]	USA	Case–control—505 cases vs. 443 controls	Alpha-track radon detectors	No overall association- RR = 1.02 (95% CI 0.5–2.0)
Steinbuch et al. [32]	USA	Case–control—173 cases vs. 254 controls	Alpha-track detectors in homes for 1 year	No clear association (Adjusted OR = 1.1 (95% CI 0.6–2.0)
Kaletsch et al. [33]	Germany	Case–control—204 leukaemia cases (plus other cancers)/~613 controls, redesigned to 82 cases vs. 209 controls	Residential histories + radon measurements for subsets	No association; with inconsistent overall effect. Perhaps small sample size
UKCCS Investigators [34]	United Kingdom	Large case–control—3177 cases vs. 3773 controls	Short-term radon measurements with questionnaires	No evidence of increased risk
Raaschou-Nielsen et al. [35]	Denmark	Case–control—2400 cases (leukaemia, CNS, lymphoma) vs. 6697 controls	Modelled cumulative radon exposure	Positive association- rate ratios of 1.63 for high cumulative exposure
Kendall et al. [36]	United Kingdom	Record-based case–control—27,447 cases vs. 36,793 controls	Predictive radon map (based on >400,000 measurements	12% ERR for ɤ and leukaemia but no significant association with radon exposures
Del Risco Kollerud et al. [37]	Norway	Cohort—712,674 children followed (1967–2009)	Geo-coded & assigned radon exposures	No association found for childhood leukaemia overall
Hauri et al. [15]	Switzerland	Prospective census-based cohort- 997 childhood leukaemia cases	Linked national radon-prediction maps to residence at the census	Radon exposure is not associated with childhood leukaemia
Peckham et al. [57]	USA	Registry-based ecologic—2147 cases (1995–2011)	Regional mean radon from the Texas Indoor Radon Survey	A marginal increase was observed, but overall inconsistency persisted; lymphoma compared with leukaemia
Nikkila et al. [38]	Finland	Exposure-prediction modelling with varying sample sizes by sub-analyses	Predicted radon concentrations for buildings with geologic predictors	Non-significant association observed
Yoshinaga et al. [39]	Japan	Cohort—preliminary report of 255 cases	Residential radon measurements	Preliminary analyses reported no evidence of association

**Table 5:** Biomarkers associated with radon exposure.

Study, Year	Location	Study Design	Sample Size	Method of Measurement	Identified Biomarkers	Study Main Findings
Taylor et al. [40]	USA	A cross-sectional study review	68 youths (aged 6–14 years)	Home radon measurement kit and biomarkers in the saliva	Salivary CRP, IL-1β, IL-6, IL-8, TNF	Multiple regression model- increased radon exposure correlates with higher levels of C-reactive protein (β = 0.31, *p* = 0.007) and interleukin-1β (β = 0.33, *p* = 0.016)
de Vocht et al. [41]	UK	ALSPAC cohort of Mothers, children and adolescents (children at birth, age 7, and age 17)	786–980 participants with complete information, depending on the sub-sample	Potential residential radon exposure estimates	Epigenetic DNA methylation at multiple ages	Radon was associated with exposure-dependent DNA methylation of cg16451995 at birth and cg01864468 at age 7
Banzon et al. [7]	USA	School-based cohort of children diagnosed with asthma, median age of 8.5	299 school children with asthma	Radon exposure (1-month average) by a spatiotemporal model	Mainly IL-5 and T_H_2-cell cytokine	Increased radon exposure (1-month average) associates with a greater increase in IL-5; 13.4%; 95% CI: 0.4–2.8; *p* = 0.044
Bilban et al. [42]	Slovenia	Case–control study of school children aged 9–12 years	85 cases exposed to radon ≥ 7000 Bq/m^3^	Annual radon doses estimated according to ICRP 65, with an outcome range of 7 to 11 mSv	Chromosomal aberrations, micronucleus assay	Increased structural chromosomal damage at a maximum of 4% among the cases, compared to 2.5% in the controls
Walczak K et al. [43]	Poland (Kowary City)	Population-based cohort analyses, including children	94 volunteers	Residential radon measured with CR39 Alpha track detector	Serum levels of phosphorylated histone gH2AX were used to correlate with DNA damage	Radon exposure is associated with increased DNA damage (gH2AX comet assay showed genotoxic effect)
Mphaga et al. [44]	South Africa	Ongoing cross-sectional study of residents near mine tailings in Gauteng	Anticipated 476 participants	Indoor radon measurement with AlphaE monitors	Results pending, could enable biomarker follow-up	Biomarker sampling not the primary goal of the study at protocol stage
Rathebe et al. [45]	Africa countries (Cameroon, Ghana, South Africa)	Multiple studies	Depending on the studies	Use of short-term vs. long-term device types; SSNTDs vs. electronic monitors	Exposure reviews	Reviews concluded that there are minimal epidemiologic/biomarker studies in Africa and called for biomonitoring research
Tunisia workplace radon surveys [46]	Tunisia	Cross-sectional study	Depends on the location; 110 locations	SSNTD measurements at workplaces (schools, universities, spas, factories)	Exposure assessment across workplaces	No biomarker data reported

## Data Availability

No new data were created or analysed in this study.

## Supplementary Materials

The following supporting information can be downloaded at: https://www.mdpi.com/article/10.3390/children13020208/s1, S1-PRISMA Checklist.
